# Integrated analysis of long non-coding RNAs and mRNAs associated with malignant transformation of gastrointestinal stromal tumors

**DOI:** 10.1038/s41419-021-03942-y

**Published:** 2021-07-03

**Authors:** Xiaonan Yin, Yuan Yin, Lei Dai, Chaoyong Shen, Na Chen, Junshu Li, Zhaolun Cai, Zhiyuan Jiang, Jian Wang, Zhou Zhao, Xin Chen, Hongxin Deng, Bo Zhang

**Affiliations:** 1 Department of Gastrointestinal Surgery, West China Hospital, Sichuan University, Chengdu, Sichuan 610041 China; 2 State Key Laboratory of Biotherapy and Cancer Center, West China Hospital, Sichuan University and Collaborative Innovation Center for Biotherapy, Chengdu, Sichuan 610041 China

**Keywords:** Sarcoma, Long non-coding RNAs

## Abstract

Malignant transformation of gastrointestinal stromal tumors (GISTs) is correlated with poor prognosis; however, the underlying biological mechanism is not well understood. In the present study, low-risk (LR) GISTs, GISTs categorized as high-risk based on tumor size (HBS), and on mitotic rate (HBM) were collected for RNA sequencing. Candidate hub lncRNAs were selected by Oncomine analysis. Expression of a selected hub lncRNA, DNM3OS, and its correlation with patients’ prognosis were analyzed using FISH staining, followed with the determination of function and underlying mechanism. Our results revealed a series of key pathways and hub lncRNAs involved in the malignant transformation of GISTs. Oncomine analysis revealed a tight association between clinical signatures and DNM3OS and suggested that DNM3OS is a hub lncRNA that is involved in the Hippo signaling pathway. In addition, DNM3OS was upregulated in HBS, HBM, and HBS/M GIST and correlated with worse prognosis in patients with GISTs. In addition, DNM3OS promoted GIST cell proliferation and mitosis by regulating the expression of GLUT4 and CD36. Collectively, these results improve our understanding of the malignant transformation of GISTs and unveil a series of hub lncRNAs in GISTs.

## Introduction

Gastrointestinal stromal tumors (GISTs), relatively rare mesenchymal disease, are the most common human sarcoma of the gastrointestinal tract, with an estimated annual incidence of 10–15 per million [1]. GISTs originate from the intestinal cells of Cajal or their precursors and are mostly driven by activating mutations in *KIT* (75–80%) or *PDGFRA* (5–10%) [2, 3]. Contrary to the oncogenic drivers of many other tumor types, gain-of-function mutations in *KIT* or *PDGFRA* are not correlated with the malignant potential or disease progression of GISTs [4, 5]. Therefore, it is believed that there are additional molecular mechanisms involved in the malignant character of GISTs.

Predicting the malignant potential of GISTs is often difficult, and substantial research has been conducted to identify potential predictive markers of GIST malignancy. Several risk stratification criteria have been applied to assess the malignant potential of GISTs, which are mainly based on clinicopathological parameters, such as tumor location, mitotic rate, and tumor size [6–9]. For GISTs at the same location, tumor size and mitotic rate are crucial indicators of malignancy. The metastasis rate for gastric GISTs ≤2 cm and a mitotic rate ≤5 mitoses/50 high-power fields (HPFs) and those >10 cm and a mitotic rate >5 mitoses/50 HPFs were 0% and 86%, respectively, according to the NCCN guidelines (Version 4; 2019). Furthermore, for gastric GISTs >10 cm, the metastasis rate for tumors with a mitotic rate ≤5 mitoses/50 HPFs and those with a mitotic rate >5 mitoses/50 HPFs were 12% and 86%, respectively (NCCN guidelines, Version 4; 2019). This evidence demonstrates the importance of tumor size and the mitotic index in evaluating the malignant transformation of GISTs. However, the biological mechanism underlying the malignant transformation of GISTs still remains unclear.

Long non-coding RNAs (lncRNAs) are a heterogeneous class of transcripts longer than 200 nucleotides that have limited protein-coding potential [10, 11]. LncRNAs are involved in multiple biological processes, including transcriptional and post-transcriptional regulation of gene expression, translation, nuclear organization, and epigenetic modification [12, 13]. The dysfunction of various lncRNAs has been shown to be strongly associated with diverse pathological conditions. In recent years, lncRNAs have been shown to be involved in cancer development by regulating cell proliferation, apoptosis, invasion, migration, stemness maintenance, and metabolism [14–16]. Several classical lncRNAs identified in other solid tumors, such as H19 and HOTAIR, were found to be differently expressed in GISTs and were correlated with high-risk grade, metastasis, and tumor progression [17, 18]. However, few studies have investigated the expression profiles of lncRNAs involved in the malignant transformation of GISTs.

In the present study, we aimed to investigate the expression profiles of lncRNAs and mRNAs associated with the malignant transformation of GISTs with an aim to identify new potential prognostic biomarkers and therapeutic targets. We performed rRNA-depleted RNA-seq analysis of gastric GIST tissues and paired adjacent normal tissues, including paired low-risk (LR) GISTs, paired high-risk GISTs based on tumor size (HBS), and paired high-risk GISTs based on mitotic rate (HBM), according to the modified NIH risk classification. Then, the co-upregulated lncRNAs in HBS and HBM were subjected to KEGG analysis. The candidate hub lncRNAs were selected by Oncomine analysis and correlation analysis, and their potential functions and underlying molecular mechanism were investigated. This work presents valuable information about the malignant transformation of GISTs and provides numerous candidate prognostic biomarkers and therapeutic targets.

## Materials and methods

### Patients and tumor samples

To construct the RNA sequencing data for GISTs, matched pairs of frozen normal and tumor tissues were collected from 10 patients (3 paired low-risk GISTs [LR, tumor size 2–5 cm and mitotic rate ≤5 mitoses/50 HPFs], 3 paired high-risk GISTs based on tumor size [HBS, tumor size >10 cm and mitotic rate ≤5 mitoses/50 HPFs], and 4 paired high-risk GISTs based on mitotic rate [HBM, tumor size 2–5 cm and mitotic rate >10 mitoses/50 HPFs]). Additionally, frozen samples from 251 patients with GIST were obtained from the Biological Specimen Banks (West China Hospital, Sichuan University, China). All these patients were diagnosed with GIST by two independent pathologists according to Chinese consensus guidelines for the diagnosis and management of GISTs. Of the 251 cases, 50 had tumors with paired adjacent normal tissues and 201 had only tumor samples. The clinicopathological parameters and follow-up data, including age at diagnosis, sex, tumor size, mitotic count, National Institutes of Health risk classifications, KIT/platelet-derived growth factor receptor-α mutational status, and distant metastasis, were extracted from the patients’ medical records. The study protocol was approved by the Research Ethics Board of West China Hospital, Sichuan University, China [Number: 2019 (1135)]. Written informed consent was obtained from each patient.

### Cell culture and lentiviral transduction

The GIST-882 cells were purchased from the Shanghai Cancer Institute (Shanghai, China). The GIST-T1 cells were kindly provided by Dr. Jian Li at Peking University Cancer Hospital and Institute (Beijing, China). The GIST-882 cells were cultured in Dulbecco’s modified Eagle’s medium (DMEM, Gibco, USA) supplemented with 10% fetal bovine serum (FBS, Gibco) and 1% penicillin/streptomycin (Gibco, USA). The GIST-T1 cells were cultured in Iscove’s modified Dulbecco Medium (IMDM; Gibco, USA) supplemented 20% FBS (Gibco). All the cell lines were maintained in a humidified 37 °C incubator with 5% CO_2_. Two shRNAs for DNM3OS (shDNM3OS 1020: 5′-CACCGCACCGACCCACAACTTATTGTTCAAGAGACAATAAGTTGTGGGTCGGTGCTTTTTTG-3′; shDNM3OS 1341: 5′-CACCGACCCTCAAGCTGAATGAAATCTTCAAGAGAGATTTCATTCAGCTTGAGGGTTTTTTTG-3′) were inserted into the GV115 lentiviral vector (GeneChem Co. Ltd, Shanghai, China), which encoded enhanced green fluorescent protein under the control of the cytomegalovirus (CMV) promoter. A non-silencing shRNA was designed as a negative control (Lv-shNC group). For DNM3OS knockdown, GIST cells were infected with lenti-shDNM3OS or lenti-non-targeting control for 72 h, as recommended by the manufacturer. The knockdown efficacy of DNM3OS was determined using quantitative polymerase chain reaction (qPCR) 5 days after infection. All infection experiments were performed independently three times.

### Cell viability assay

Cell viability was measured using the Cell Counting Kit-8 (CCK-8) assay. Briefly, cells from each group were inoculated into 96-well plates (1000 cells/well) in 100 μL of DMEM containing 10% FBS. Then, 10 μL of CCK-8 reagent (Dojindo, Kumamoto, Japan) was added to each well and incubated for 0, 24, 48, and 72 h according to the manufacturer’s instructions. Absorbance at 450 nm was measured using a microplate reader (MK3, Thermo Fisher, MA, USA).

### RNA isolation, library construction, and RNA sequencing

Total RNA was extracted using TRIzol (Invitrogen, MA, USA) following the manufacturer’s protocol. It was purified and then quantified using a NanoDrop 2000 spectrophotometer (Thermo Fisher Scientific, Waltham, MA, USA). RNA integrity was evaluated using an Agilent 2100 Bioanalyzer (Agilent Technologies, Santa Clara, CA, USA). Next, the libraries were constructed using TruSeq Stranded Total RNA with Ribo-Zero Gold (Illumina, Cat. RS-122–2301) according to the manufacturer’s instructions. Then, the libraries were sequenced using an Illumina Hiseq X Ten Platform and 150 bp paired-end reads were generated. Lastly, all sequencing processes were conducted by OE Biotech Co., Ltd (Shanghai, China). The significantly differentially expressed lncRNAs were screened based on the fold change >2 at a *p* value < 0.05.

### Pathway enrichment analysis

Significantly differentially expressed lncRNAs among groups were selected to perform pathway enrichment analysis using the Kyoto Encyclopedia of Genes and Genomes (KEGG) pathway analysis [19]. Differences were considered significant at *p* value < 0.05.

### Oncomine database analysis

Based on the expression profile in GISTs (LR, HBS, and HBM groups), the top 150 genes that positively and negatively correlated with lncRNAs H19, DNM3OS, DPP10-AS1, PRKCQ-AS1, AC010980.2, SOCS2-AS1, FENDRR, IGF2-AS, MRPL23-AS1, LINCO01096, and MEIS1-AS3 were selected. The coding genes were used for Oncomine analysis based on the common expression between selected genes and gene clusters that demonstrated the clinical signatures of gastric cancer. The analyses were performed using Oncomine gene expression array datasets (www.oncomine.org).

### Protein–protein interaction (PPI) analysis based on String database

The correlation between DNM3OS expression and GLUT4 and CD36 expression was analyzed based on the sequencing data of GIST-882 cells. PPI analyses were performed using the String database (https://string-db.org/cgi/input.pl).

### Quantitative PCR analysis

Total RNA was extracted using TRIzol (Invitrogen) and the reverse transcription reaction was performed using the PrimeScript RT Reagent Kit (Perfect Real Time, Takara, Tokyo, Japan), according to the manufacturer’s instructions. Additionally, the nuclear and cytoplasmic RNAs from GIST-882 cells were separated using a PARIS kit (AM1921; Thermo Fisher Scientific), following the manufacturer’s protocol. To determine the relative expression of RNAs, qPCR was performed using TB Green Premix Ex TaqTM II (Tli RNaseH Plus, Takara). For all qPCRs, β-actin was measured as an internal reference. qPCR was performed as follows: 95 °C for 30 s for pre-denaturation, 95 °C for 5 s, and 58 °C for 30 s for 42 cycles. A final dissociation curve was obtained. The primer sequences are listed as follows: GDF11 sense 5′-AACTT CCCCAGATACCCCGT‐3′, GDF11 anti-sense 5′‐GGGTGGGTAGAGCAATCAGG‐3′; TNXB sense 5′‐CAGCCCAGTATGCTCTAACC‐3′, TNXB anti-sense 5′‐ATT GGACCGTGAAGAGAAGGG‐3′; FP248 sense 5′‐CCTCCCAACATAACCTCCT‐3′, FP248 anti-sense 5′‐CTGACCATTCATCCCGATA‐3′; TCONS_00023602 sense 5′‐GGAAGCGGAGGAGAGTAAAG‐3′, TCONS_00023602 anti-sense 5′‐GGGAT TAACTGGAGCCTATCAC‐3′; DNM3OS sense 5′‐GTCTCATTCTGGGAGCTG TC‐3′, DNM3OS anti-sense 5′‐AATGCCTTGTACCACCTGTT‐3′; ENSG00000137225 sense 5′‐GTGTGACCAGGACCATTCAGG‐3′, ENSG00000137225 anti-sense 5′‐TCAGCTTGATGCCTGCTTTCT‐3′; ENSG00000167483 sense 5′‐TGT CAAGTTTCCTGGCTGGG‐3′, ENSG00000167483 anti-sense 5′‐GCTGAACCAA GAACACACGG‐3′.

### Fluorescence in situ hybridization

RNA fluorescence in situ hybridization (FISH) was performed using the DNM3OS probe, as previously described [20]. To detect RNA-FISH in formalin-fixed paraffin-embedded tissues, paraffin-embedded tissue sections were de-paraffinized in xylene, rehydrated in a series of graded alcohols, and digested with pepsin. After prehybridization with the prehybridization buffer for 2 h at 42 °C, the sections were incubated with the hybridized probes in hybridization buffer for 5 min at 83 °C and overnight at 42 °C. The sections were washed with saline sodium citrate (SSC) solution, followed by nuclear staining with 4′,6′-diamidino-2-phenylindole (DAPI; Thermo Fisher). For RNA-FISH in GIST cells, coverslip-grown cell samples were fixed with 4% paraformaldehyde at room temperature for 10 min. After washing the cells with phosphate-buffered saline (PBS), the samples were permeabilized with 0.5% Triton X-100 for 5 min at 4 °C. After washing, the samples were incubated with the DNM3OS probe in hybridization solution overnight at 37 °C. After washing the cells with SSC solution, nuclei were stained with DAPI. Images were obtained using a fluorescence microscope (Olympus, Japan). The expression of DNM3OS in paraffin-embedded tissue sections was scored as 0 (negative), 1 (weak staining), 2 (strong staining in less than 30% of tumor cells), and 3 (strong staining in more than 30% of tumor cells). For each section, three fields were randomly selected and the score of DNM3OS expression was calculated by experienced researchers. A final score was calculated as the sum of the three scores from 0 to 9. The median score for DNM3OS expression was 4; tumors with a total score exceeding 4 were defined as high expressers of DNM3OS; otherwise, they were considered to have a low expression of DNM3OS.

### Immunofluorescence

For the immunofluorescence assay, cells were washed with PBS and fixed with 4% paraformaldehyde at room temperature for 10 min. After washing, the cells were permeabilized with 0.1% Triton X for 10 min at room temperature. The cells were then incubated with phospho-histone H3 (Ser10) antibody (1:100 dilution; Cell Signaling Technology) at 4 °C overnight. Then, the cells were incubated with Alexa Fluor 568-conjugated secondary antibody (1:100 dilution, Invitrogen, USA) for 1 h at 37 °C. After washing the cells with PBS, nuclei staining was performed with DAPI for 10 min at room temperature. Images were captured using a fluorescence microscope (Olympus).

### Western blotting

Total cell protein was isolated using radioimmunoprecipitation lysis buffer (Beyotime, Beijing, China) in the presence of protease and phosphatase inhibitors (Beyotime). Proteins were separated using sodium dodecyl sulfate-polyacrylamide gel electrophoresis (SDS-PAGE) and transferred to nitrocellulose membranes. After blocking with Tris-buffered saline/0.1% Tween 20 (TBS/T) buffer containing 5% non-fat milk, the blots were incubated with primary antibodies for 1.5 h at room temperature. Next, the blots were incubated with horseradish peroxidase-conjugated secondary antibodies for 1 h at room temperature. Proteins were examined using enhanced chemiluminescence (Thermo Scientific). The primary antibodies used were anti-CD36 (1:1000, 18836-1-AP; Proteintech) and anti-GLUT4 (1:3000, 66846-1-Ig; Proteintech).

### Statistical analysis

SPSS (version 21.0 for Windows, SPSS Inc., Chicago, IL, USA) and GraphPad Prism 7 (GraphPad Prism 7.0, San Diego, CA, USA) were used for statistical analyses. Quantitative variables are presented as mean ± standard deviation (SD) and were analyzed using the Student’s *t*-test or one-way analysis of variance. Categorical variables are expressed as percentages and statistical significance was tested using the Chi-squared or Fisher’s exact tests. Kaplan–Meier curves and the log-rank test were used to compare survival times among groups. A *p* value < 0.05 was considered statistically significant.

## Results

### Sequencing and screening of hub lncRNAs involved in malignant transformation of GISTs

**Fig. 1:**
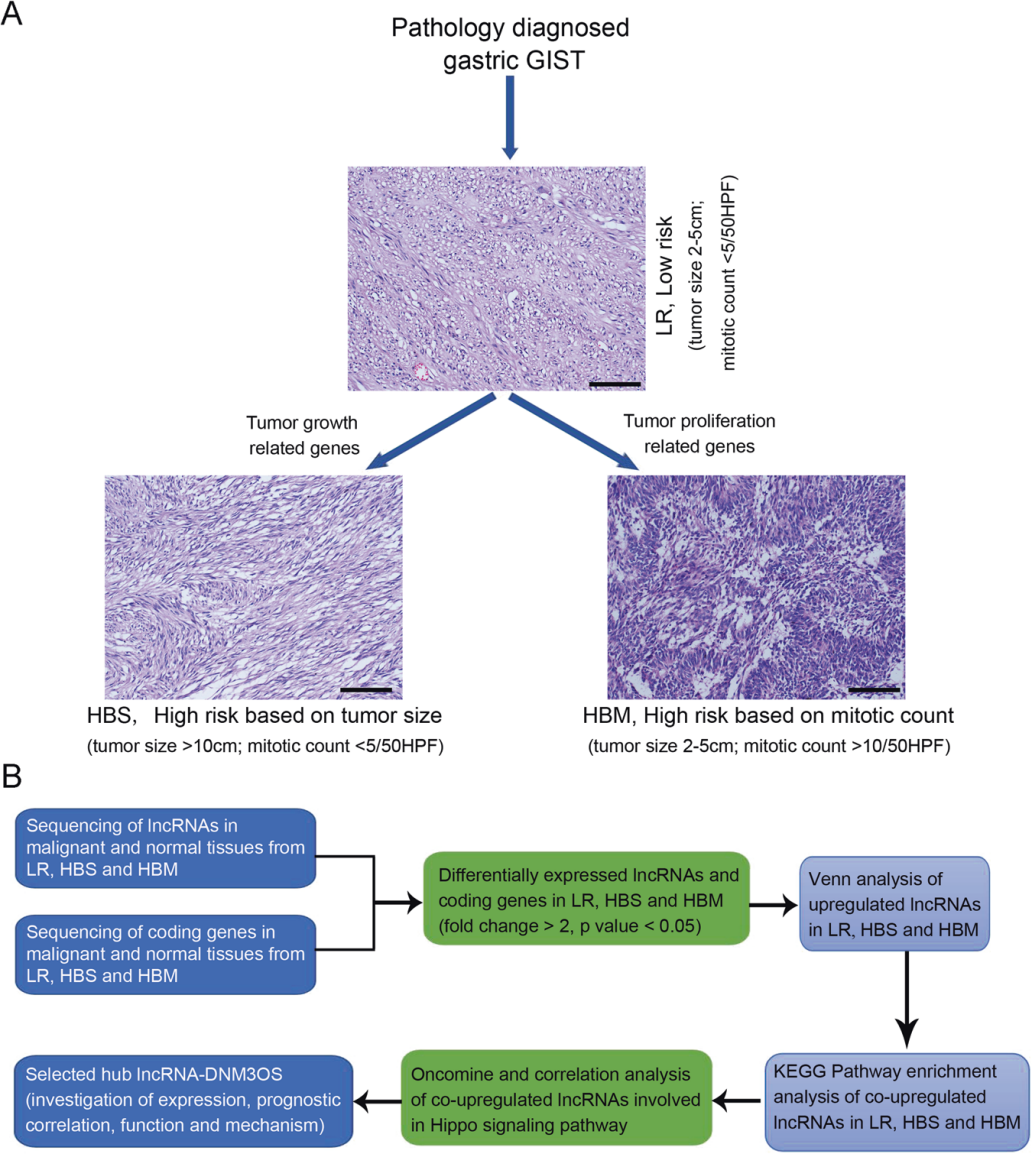
Workflow for this study. **A** Clinicopathological features of the selected GIST cases for RNA sequencing. **B** Comprehensive analyses of mRNA and lncRNA in GISTs. GIST gastrointestinal stromal tumor, lncRNA long non-coding RNA.

To screen the hub lncRNAs expressed during GIST malignant transformation, 10 pairs of malignant gastric GISTs with different clinical risk classifications were collected along with adjacent normal tissues for RNA sequencing. The samples included three paired LR GISTs (tumor size 2–5 cm and mitotic rate ≤5 mitoses/50 HPFs), three paired HBS GISTs (tumor size >10 cm and mitotic rate ≤5 mitoses/50 HPFs), and four paired HBM GISTs (tumor size 2–5 cm, and mitotic rate >10 mitoses/50 HPFs). The screening criteria and detailed clinicopathological features of the 10 sequencing samples are listed in Fig. 1A and Supplemental Table S1, respectively. After sequencing, the significantly differentially expressed lncRNAs and mRNAs between malignant and normal tissues were selected based on the fold change and *p* value and then subjected to Venn analysis to screen for co-upregulated lncRNAs in HBS and HBM (Fig. 1B). KEGG pathway enrichment analysis, Oncomine, and correlation analysis were employed to identify the hub lncRNAs in the Hippo signaling pathway (Fig. 1B). Finally, the expression, prognostic correlation, function, and mechanism of one candidate hub lncRNA, DNM3OS, were investigated in GIST cells (Fig. 1B).

### Expression profiles of lncRNAs during malignant transformation of GISTs

**Fig. 2:**
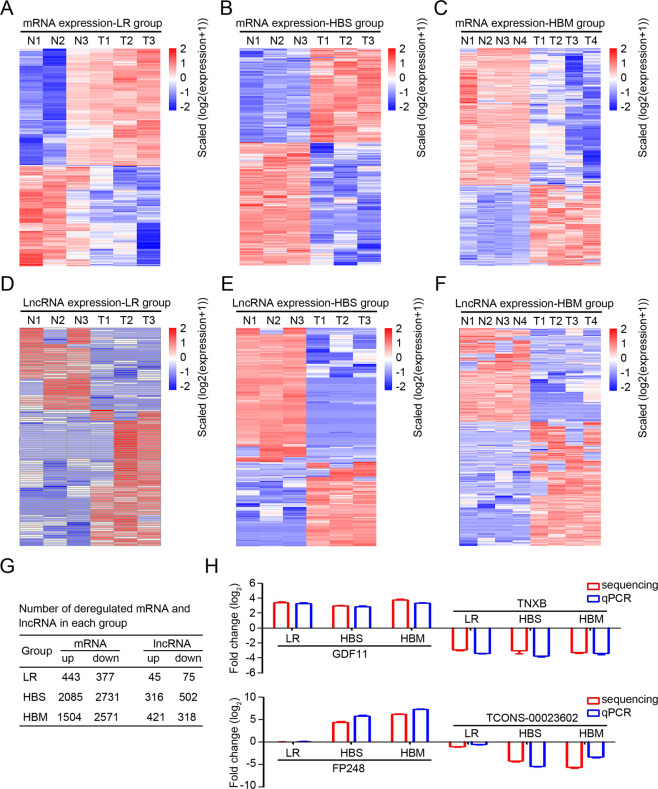
Expression profiles of mRNA and lncRNA in GIST and corresponding adjacent normal tissue. **A**–**C** Heatmap of differential expression of mRNA among (**A**) low-risk (LR) GIST and control, **B** high-risk GIST based on tumor size (HBS) and control, and (**C**) high-risk GIST based on mitotic rate (HBM) and control. **D**–**F** Heatmap of differential expression of lncRNAs among (**D**) LR GIST and control, (**E**) HBS GIST and control, and (**F**) HBM GIST and control. **G** List and number of deregulated mRNAs and lncRNAs in LR, HBS, and HBM GIST. **H** Two randomly selected coding genes (GDF11 and TNXB) and two randomly selected lncRNAs (FP248 and TCONS-00023602) were selected for qPCR. Fold change of coding genes determined by sequencing and qPCR are displayed. GIST gastrointestinal stromal tumor, lncRNA long non-coding RNA, qPCR quantitative polymerase chain reaction.

To better analyze the potential function of lncRNAs in the malignant transformation of GISTs, the expression profiles of coding genes and lncRNAs were analyzed by RNA sequencing. The expression profiles of all differentially expressed mRNAs in the LR (Fig. 2A), HBS (Fig. 2B), and HBM groups (Fig. 2C) were displayed using heatmap plots. The lncRNA expression profiles are also displayed in Fig. 2D–F. The results showed that 820 mRNAs (443 upregulated and 377 downregulated) were deregulated in LR GISTs, 4816 mRNAs (2085 upregulated and 2731 downregulated) were deregulated in HBS GISTs, and 4075 mRNAs (1504 upregulated and 2571 downregulated) were deregulated in HBM GISTs when compared with the corresponding levels in paired adjacent normal tissues (Fig. 2G). In addition, 120 lncRNAs (45 upregulated and 75 downregulated) were deregulated in LR GISTs, 818 lncRNAs (316 upregulated and 502 downregulated) were deregulated in HBS GISTs, and 739 lncRNAs (421 upregulated and 318 downregulated) were deregulated in HBM GISTs when compared with the corresponding levels in paired adjacent normal tissues (Fig. 2G). To confirm the accuracy of the sequencing data, two random mRNAs (GDF11 and TNXB) and two random lncRNAs (FP248 and TCONS-00023602) were selected for qPCR determination. We confirmed that the expression trends of GDF11, TNXB, FP248, and TCONS-00023602 as detected by qPCR verified the RNA sequencing results (Fig. 2H). We performed KEGG pathway analysis of the upregulated and downregulated lncRNAs to identify possible pathways involved in GIST malignant transformation. The top 20 pathways for LR, HBS, and HBM are shown in Supplementary Fig. 1. These results revealed the expression profiles of lncRNAs in GISTs and their potential functions.

### Functional annotation of the co-upregulated lncRNAs in HBS and HBM GISTs

**Fig. 3:**
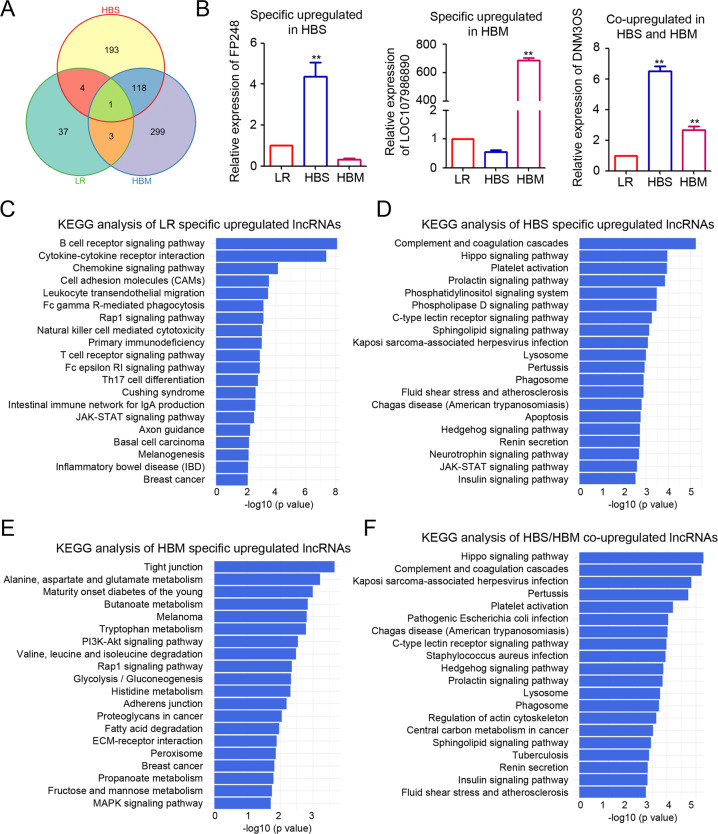
KEGG pathway analysis of differentially expressed lncRNAs. **A** Venn diagram showing the total count of shared and specific lncRNAs among low-risk (LR) GIST, high-risk GIST based on tumor size (HBS), and high-risk GIST based on mitotic rate (HBM). **B** Three randomly selected lncRNAs (FP248, LOC107986890, and DNM3OS) were selected for qPCR. **C**–**F** KEGG pathway analysis of significantly upregulated lncRNAs in LR (**C**), HBS (**D**), HBM (**E**), and HBS/HBM (**F**). GIST gastrointestinal stromal tumor, KEGG Kyoto Encyclopedia of Genes and Genomes, lncRNA long non-coding RNA, qPCR quantitative polymerase chain reaction.

To determine the predictive roles of the identified lncRNAs in the malignant transformation of GISTs, Venn diagram analysis was performed to distinguish the lncRNAs specifically upregulated in LR, HBS, and HBM GISTs, which identified 37, 193, and 299 specifically upregulated lncRNAs, respectively (Fig. 3A). The intersection between the lncRNAs upregulated in HBS and HBM GISTs revealed 118 lncRNAs co-upregulated in both HBS and HBM GISTs (Fig. 3A). The expression profiles of the co-upregulated lncRNAs in HBS and HBM were displayed using a heatmap plot (Supplementary Fig. 2). Three random lncRNAs, FP248, LOC107986890, and DNM3OS, were selected for qPCR analysis, and their expression trends were consistent with the RNA sequencing results (Fig. 3B). Next, KEGG analysis was performed to identify the possible pathways containing the specific upregulated lncRNAs involved in GIST malignant transformation. The lncRNAs specifically upregulated in LR GISTs were found to be involved in B cell receptor signaling pathway, cytokine–cytokine receptor interaction, and chemokine signaling pathway (Fig. 3C). The lncRNAs specifically upregulated in HBS GISTs were found to be involved in complement and coagulation cascades, the Hippo signaling pathway, and platelet activation (Fig. 3D). The main pathways for the lncRNAs specifically upregulated in HBM GISTs included tight junction, alanine aspartate, and glutamate metabolism and maturity onset diabetes of the young (Fig. 3E). Notably, the co-upregulated lncRNAs in HBS and HBM GISTs appear to be involved in the Hippo signaling pathway, complement and coagulation cascades, and Kaposi sarcoma-associated herpesvirus infection (Fig. 3F). These results suggest that several signaling pathways, especially the Hippo signaling pathway, may be involved in the malignant transformation of GISTs.

### Screening of hub lncRNAs in malignant transformation of GISTs by Oncomine analysis

**Fig. 4:**
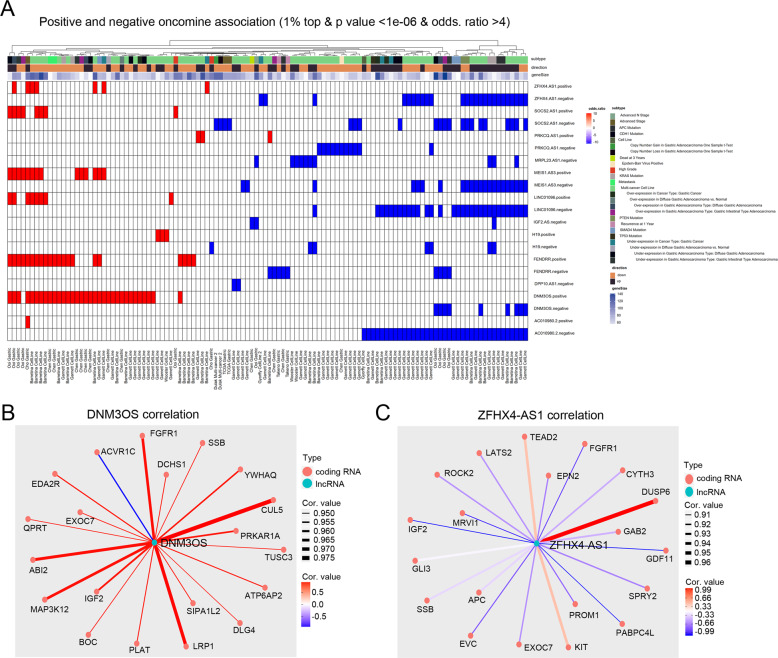
Oncomine analysis of co-upregulated lncRNAs in high-risk GIST based on tumor size (HBS) and high-risk GIST based on mitotic rate (HBM) involved in the Hippo signaling pathway. **A** Top 150 genes that positively and negatively correlated with 12 lncRNAs were selected for Oncomine analysis. After mapping with the gene clusters, the heatmap displayed the top 1% overlap between genes correlated to lncRNAs and genes positively associated with various gastric cancer clinical signatures. **B** Correlation between DNM3OS and top 20 coding genes. **C** Correlation between ZFHX4-AS1 and top 20 coding genes. lncRNA, long non-coding RNA.

To investigate the potentially oncogenic lncRNAs involved in the growth and proliferation of GISTs, we focused on the lncRNAs involved in the most relevant signaling pathway identified by KEGG analysis of HBS/HBM co-upregulated lncRNAs, the Hippo signaling pathway. The analysis identified 45 upregulated lncRNAs involved in the Hippo signaling pathway, and 12 clinically relevant lncRNAs were selected for further Oncomine analysis. The expression details of the 12 lncRNAs are presented in Supplementary Table 2. Owing to the limited lncRNA expression data in the Oncomine database, the top 150 coding genes correlated with the expression of the lncRNAs (positive and negative) were used in the Oncomine analysis. After mapping the gene clusters identified in the Oncomine database, the top 1% of overlap between the top 150 genes correlated with the lncRNAs, and the genes positively associated with various gastric cancer clinical signatures were displayed as a heatmap (Fig. 4A). Genes that were positively correlated with DNM3OS were significantly associated with clinical signatures related to high grade, metastasis, and gene mutations, and genes negatively correlated with ZFHX4-AS1 were significantly associated with clinical signatures related to gene mutations (Fig. 4A and Supplemental Fig. 3). The top 20 coding genes that were positively or negatively correlated with DNM3OS and ZFHX4-AS1 are listed in Fig. 4B and C, respectively. Based on the tight correlation between DNM3OS and the clinical signatures of gastric cancer, we selected DNM3OS for further investigation.

### Overexpression of DNM3OS is correlated with worse prognosis in patients with GISTs

**Fig. 5:**
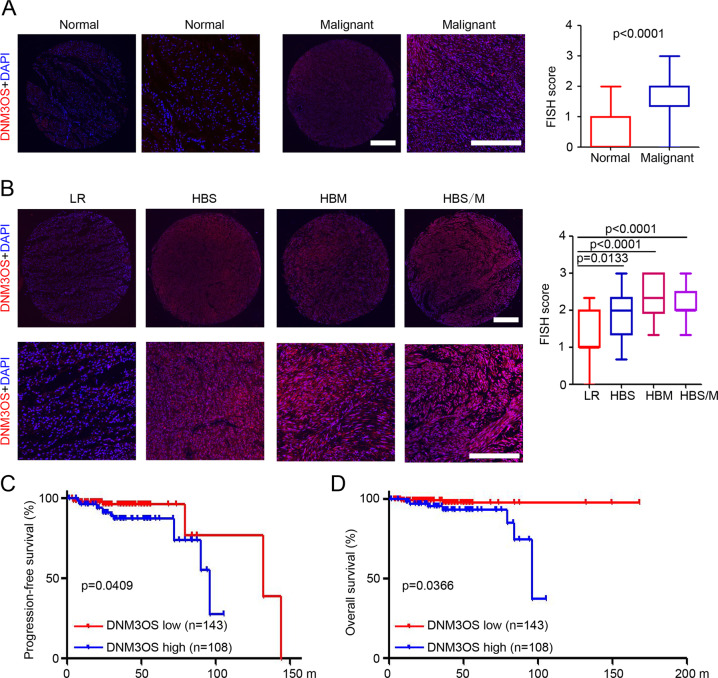
DNM3OS expression was upregulated in GISTs and correlated with poor prognosis in patients with GIST. **A** One representative result of DNM3OS expression in GIST tissues compared to paired normal tissues using FISH. DNM3OS expression between normal and malignant cells was analyzed based on the FISH score. Scale bar = 200 μm. **B** FISH staining of DNM3OS expression in low-risk (LR) GIST, high-risk GIST based on tumor size (HBS), high-risk GIST based on mitotic rate (HBM), and high-risk GIST based on tumor size and mitotic rate (HBS/M). DNM3OS expression among these groups was analyzed based on the FISH score. Scale bar = 200 μm. **C** Kaplan–Meier analysis of the progression-free survival of the 251 patients with GIST based on DNM3OS expression. **D** Kaplan–Meier analysis of the overall survival of 251 patients with GIST based on DNM3OS expression. GIST gastrointestinal stromal tumor, FISH fluorescence in situ hybridization, lncRNA long non-coding RNA.

To determine the expression and potential correlation between DNM3OS expression and the prognosis of patients with GISTs, FISH staining was performed on three tissue microarrays containing 251 GISTs and corresponding adjacent normal tissues. The results demonstrated that the expression of DNM3OS was significantly elevated in the GISTs when compared to the levels in adjacent normal tissues (Fig. 5A). The 251 patients were divided into LR, HBS, HBM, and HBS/M (tumor size >10 cm and mitotic rate >10 mitoses/50 HPFs) groups. The results indicated that DNM3OS expression was markedly higher in HBS and HBM GISTs than in LR GISTs (Fig. 5B). Further analysis also suggested that DNM3OS is upregulated in HBS/M GISTs (Fig. 5B). Based on the median DNM3OS expression level, the 251 patients with GISTs were divided into high (108 patients) and low DNM3OS groups (143 patients). Then, the correlation between DNM3OS expression and various clinicopathological parameters was analyzed. The results indicated that DNM3OS expression was positively associated with tumor size, mitotic count, NIH risk classification, and mutational status (Supplementary Table 3). Moreover, Kaplan–Meier analyses revealed that higher DNM3OS expression was associated with shorter progression-free survival (PFS) and overall survival (OS) (Fig. 5C, D). Collectively, these findings demonstrated that DNM3OS was upregulated in GIST tissues and was associated with poor patient prognosis.

### DNM3OS knockdown suppresses GIST cell proliferation and mitosis

**Fig. 6:**
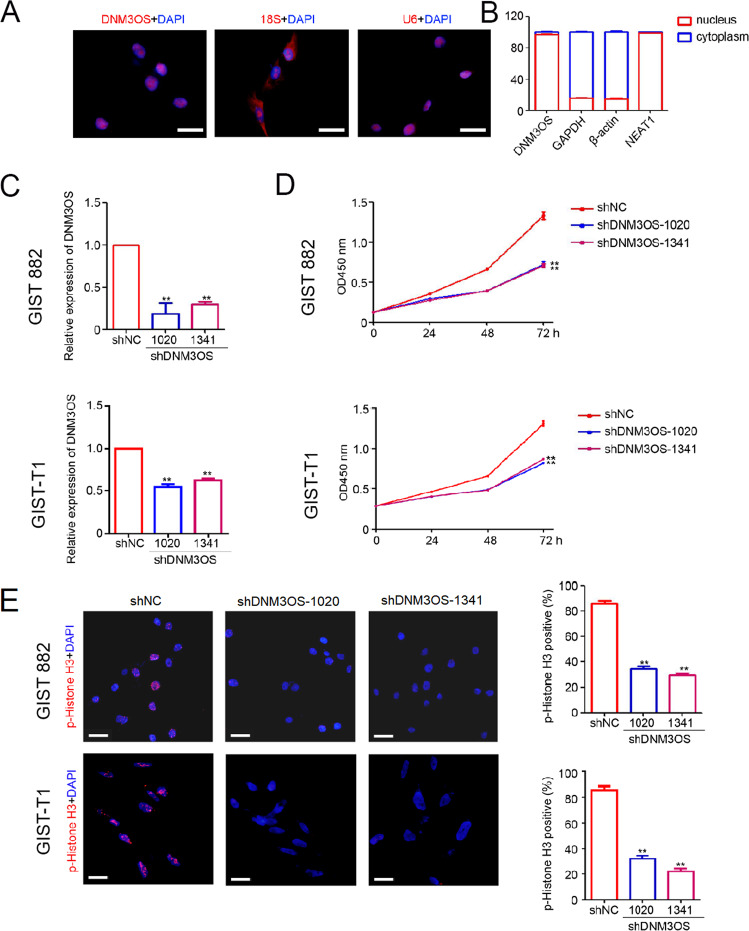
DNM3OS knockdown suppressed the proliferation and mitotic index of GIST cells in vitro. **A** Staining of DNM3OS by FISH in GIST-882 cells (red). Nuclear RNA U6 snRNA and cytoplasmic 18S snRNA served as controls. Scale bar = 20 μm. **B** Nuclear and cytoplasmic RNAs were extracted for DNM3OS determination using qPCR. GAPDH, β-actin, and NEAT1 were used as controls. **C** qPCR was employed to determine the expression of DNM3OS that was stably infected with lenti-shNC, lenti-shDNM3OS-1020, and lenti-shDNM3OS-1341. **D** Viability of GIST-882 cells and GIST-T1 cells were detected using the CCK-8 assay (*n* = 5, **p < 0.01). **E** Immunofluorescence analysis of p-histone H3 expression in GIST-882 cells and GIST-T1 cells. Percentage of p-histone H3positive cells in each frame was analyzed (*n* = 5, **p < 0.01). CCK-8 Cell Counting Kit-8, GIST gastrointestinal stromal tumor, FISH fluorescence in situ hybridization, lncRNA long non-coding RNA, qPCR quantitative polymerase chain reaction, snRNA small nuclear RNA.

To investigate the potential biological role of DNM3OS in GIST cells, we determined the location of DNM3OS in GIST-882 cells by FISH staining. As shown in Fig. 6A, DNM3OS was mainly located in the nucleus. qPCR results for specific RNAs from the nucleus and cytoplasm also suggested that most DNM3OS was expressed in the nucleus (Fig. 6B). Next, GIST-882 cells and GIST-T1 cells were infected with lentivirus-mediated shRNAs targeting DNM3OS, and analysis showed significant downregulation of DNM3OS in GIST-882 cells and GIST-T1 cells stably infected with lenti-shDNM3OS-1020 and lenti-shDNM3OS-1341 (Fig. 6C). To clarify the role of DNM3OS in GIST cell proliferation, the CCK-8 assay was performed, and the results showed that DNM3OS knockdown significantly inhibited cell proliferation (Fig. 6D). To evaluate the effects of DNM3OS expression on the mitotic index, p-histone H3 immunofluorescence was evaluated. The results showed that the mitotic index was significantly reduced in DNM3OS-knockdown cells (Fig. 6E). These data indicate that DNM3OS promotes GIST cell proliferation and mitosis.

### DNM3OS regulates several pathways by promoting the expression of GLUT4 and CD36

**Fig. 7:**
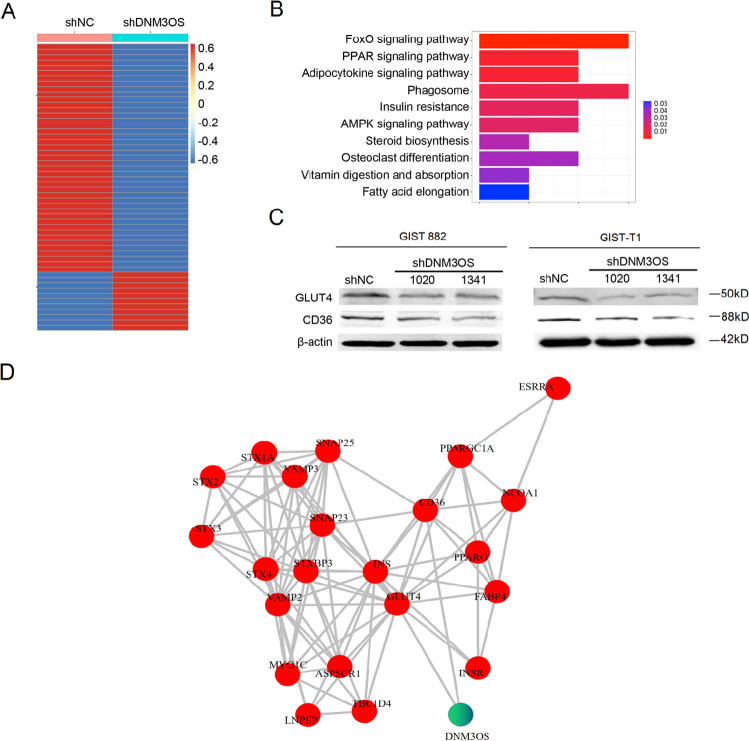
DNM3OS regulated several pathways via promotion of GLUT4 and CD36 expression. **A** Hierarchical clustering of coding genes altered after knockdown of DNM3OS in GIST-882 cells. **B** KEGG analysis of deregulated genes in GIST-882-shDNM3OS cells. **C** Western blot assay showing the expression of GLUT4 and CD36 in GIST-882 cells and GIST-T1 cells. GAPDH was used as the loading control. **D** Protein–protein interaction analysis of DNM3OS-regulated proteins that are linked by GLUT4 and CD36. GIST gastrointestinal stromal tumor, KEGG Kyoto Encyclopedia of Genes and Genomes.

To investigate the mechanism by which DNM3OS regulates GIST cell proliferation and mitosis, GIST-882-shNC and GIST-882-shDNM3OS cells were collected for RNA sequencing. The results showed that in shDNM3OS cells, 11 coding genes were upregulated and 87 coding genes were downregulated (Fig. 7A). The expression trends of two randomly selected genes, *CAPN11* and *FAM129C*, as examined by qPCR, verified the results from RNA sequencing (Supplemental Fig. 4). KEGG analysis suggested that the FoxO, PPAR, and AMPK signaling pathways are regulated by DNM3OS (Fig. 7B). GLUT4 and CD36, which were previously shown to be cancer oncogenes [21–24], were predicted to be downstream targets of DNM3OS. Thus, we evaluated the expression levels of GLUT4 and CD36 in GIST-882 cells and GIST-T1 cells by western blotting. The results indicated that knockdown of DNM3OS in GIST cells inhibited the expression of GLUT4 and CD36 (Fig. 7C), suggesting that they also function as oncogenes in GISTs. PPI analysis based on the String database revealed the regulatory network of DNM3OS, which is linked to GLUT4 and CD36. These results clarified the underlying mechanism by which DNM3OS regulates malignant transformation of GISTs.

## Discussion

Our study analyzed the lncRNA expression profiles in GISTs with differential malignant potential by RNA sequencing. A series of key pathways and hub genes were identified that may be differentially expressed during the malignant transformation of GISTs. Oncomine analysis showed a tight association between clinical signatures and DNM3OS and suggested that DNM3OS is a hub lncRNA that is involved in the Hippo signaling pathway. In addition, DNM3OS was upregulated in HBS, HBM, and HBS/M GISTs and was correlated with worse prognosis in patients with GISTs. DNM3OS promoted GIST cell proliferation and mitosis by regulating the expression of GLUT4 and CD36. The present study demonstrated, for the first time, the expression profiles of lncRNAs during the malignant transformation of GIST and provided a series of candidate prognostic biomarkers and therapy targets for GISTs, such as the lncRNA DNM3OS.

LncRNAs have been shown to play important roles in the regulation of various biological processes and the development of various diseases [25]. Several lncRNAs identified in other malignant tumors, such as HOTAIR, CCDC26, and AOC4P, have also been evaluated in GISTs and were shown to be associated with GIST metastasis and sensitivity to imatinib [17, 26, 27]. Gyvyte et al. [28] performed a next-generation sequencing study of paired GISTs and adjacent tissue samples from 15 patients and identified two deregulated lncRNAs (H19 and FENDRR) in GISTs. In addition, a recent study by Yan et al. [29] demonstrated differential expression of lncRNAs between imatinib mesylate-resistant GISTs and primary GISTs, which suggested that these dysregulated lncRNAs could serve as potential biomarkers or drug targets for GISTs, particularly secondary imatinib-resistant GISTs. However, no study has investigated the expression profiles of lncRNAs involved in malignant transformation of GISTs. In this study, we identified lncRNAs related to GIST malignant transformation and their potential functions. To prioritize the lncRNAs that were the most relevant to tumor proliferation and mitosis, we focused on lncRNAs that were co-upregulated in both HBS and HBM GISTs when compared to LR GISTs by Venn analysis. KEGG analysis of these lncRNAs revealed that the top three pathways were the Hippo signaling pathway, complement and coagulation cascades, and Kaposi sarcoma-associated herpesvirus infection. The Hippo signaling pathway is involved in tissue size regulation, cell proliferation, and apoptosis, and dysregulation of this pathway has a significant effect on cancer development [30–32]. However, the role of the Hippo signaling pathway in the pathogenesis of GIST has not been well studied. Qu et al. [33] found that the hippo pathway effectors YAP and TAZ could coordinately regulate cyclin D1 expression, which functions as an oncogenic mediator in KIT-independent GISTs. Our study indicated that the Hippo signaling pathway was the most relevant pathway regulated by lncRNAs involved in the proliferation and mitosis of GISTs, suggesting that the Hippo signaling pathway may play a crucial role in lncRNA-mediated regulation of malignant transformation of GISTs.

Oncomine is a widely used database for predicting the correlation between genes and clinical signatures for diverse cancers [34–36]. In the Oncomine database, one clinical signature is correlated with hundreds of coding genes and signaling pathways [37]. However, correlations between lncRNA expression and clinical signatures are limited in the Oncomine database, owing to the absence of lncRNA expression data [37]. Thus, in the present study, the top 150 genes were selected to represent the specific lncRNA, as described previously [38]. However, because of the absence of a gastric GIST database, we performed the analysis in the gastric Oncomine database, and then verified the findings in GIST tissue microarrays. Our results indicated that DNM3OS was the top-ranked lncRNA correlated with the clinical signature of gastric cancer. Further analysis demonstrated the predictive role of DNM3OS in a worse prognosis for patients with GISTs, which was consistent with the results of a previous study [39].

DNM3OS is located within an intron of human dynamin-3 (*DNM3*) and is an anti-sense transcript [40]. DNM3OS regulates gene expression post-transcriptionally, and overexpression of DNM3OS has been shown to be associated with tumor progression and radio resistance in ovarian cancer, gastric cancer, and esophageal squamous cell carcinoma [39, 41, 42]. Our results indicated that DNM3OS was higher in GISTs tissues than in adjacent normal tissues. An analysis of 251 patients with GISTs showed that high DNM3OS expression was associated with tumor size, mitotic count, NIH risk classification, PFS, and OS of patients with GISTs. These results suggest that DNM3OS might be a prognostic indicator for GISTs. To further investigate the role of DNM3OS in malignant transformation, we analyzed the effect of DNM3OS depletion on cell viability and the mitotic index. Our results demonstrated that silencing of DNM3OS in GIST cells reduced cell viability and the mitotic index. These results confirmed the role of DNM3OS as an oncogenic lncRNA in the progression of various cancers, consistent with the results of previous studies [39, 43].

Mechanistic investigations revealed that DNM3OS upregulates IGF1 expression to promote proliferation and inhibit apoptosis of CHON-001 cells by sponging miR-126 [44]. Another study suggested that DNM3OS promotes TGFβ1-induced transformation of prostate stromal cells into myofibroblasts via miR-29a/29b/COL3A1 and miR-361/TGFβ1 Axes [45]. In our study, RNA sequencing of DNM3OS knockdown cells showed that the most significantly changed pathways were the FoxO, PPAR, and adipocytokine signaling pathways. PPI analysis revealed the regulatory network of DNM3OS, which is linked to GLUT4 and CD36. However, further functional experimental studies are needed to confirm the role of DNM3OS in GISTs.

In conclusion, to our knowledge, this is the first study to evaluate the expression profiles of lncRNAs and mRNAs during the malignant transformation of GIST and identify the related biological pathways. These defined hub lncRNAs, which are involved in the Hippo signaling pathway, are a series of novel potential predictive biomarkers and therapeutic targets for GIST. Furthermore, knockdown of one hub lncRNA, DNM3OS, inhibited the proliferation and mitosis of GIST cells, and thus, it could be useful as a prognostic indicator and/or therapeutic target. These results provide a better understanding of the malignant transformation of GISTs and reveal a series of hub lncRNAs for GISTs. Further investigation is needed to verify the mechanism underlying the effects of these lncRNAs.

## Supplementary information

Supplementary Figure Legends

Supplementary Figure 1

Supplementary Figure 2

Supplementary Figure 3

Supplementary Figure 4

Supplementary Table 1

Supplementary Table 2

Supplementary Table 3

## Data Availability

RNA-seq data from this study have been deposited under the accession number PRJNA634425 at the NCBI Sequence Read Archive (SRA).
