# Supplementary material for: Integrated analysis of long non-coding RNAs and mRNAs associated with malignant transformation of gastrointestinal stromal tumors
Source: Cell Death Dis. 2021 Jul 3;12(7):669. doi: 10.1038/s41419-021-03942-y (PMC8254811; doi:10.1038/s41419-021-03942-y)
Supplement: Supplementary file 1 — Supplementary Figure Legends [file 41419_2021_3942_MOESM1_ESM.docx]

**Supplementary Figure 1.** Significantly enriched KEGG pathway of lncRNAs in the GIST subgroup. (A–F) KEGG pathway analysis of significantly deregulated lncRNAs in the LR GIST subgroup (A–B), HBS GIST subgroup (C–D), and HBM GIST subgroup (E–F).

**Supplementary Figure 2.** Hierarchical clustering of co-upregulated lncRNAs in both HBS and HBM GISTs compared with LR GISTs.

**Supplementary Figure 3.** Detailed correlation between DNM3OS and clinical signatures of gastric cancer.

**Supplementary Figure 4.** Two random coding genes (CAPN11 and FAM129C) were selected for qPCR. (A) One randomly selected downregulated coding gene (CAPN11) in the shDNM3OS group was selected for qPCR. The relative expression of coding genes determined using sequencing and qPCR is shown.(B) One randomly selected upregulated coding gene (FAM129C) in the shDNM3OS group was selected for qPCR. The relative expression of coding genes determined using sequencing and qPCR is shown.

**Supplementary Table 1.** Clinicopathological features of sequencing samples

**Supplementary Table 2.** Upregulated lncRNAs involved in the Hippo signaling pathway

**Supplementary Table 3.** Association between DNM3OS expression and clinicopathological characteristics of patients with GIST
