# Supplementary figures and images for: Integrated analysis of long non-coding RNAs and mRNAs associated with malignant transformation of gastrointestinal stromal tumors
Source: Cell Death Dis. 2021 Jul 3;12(7):669. doi: 10.1038/s41419-021-03942-y (PMC8254811; doi:10.1038/s41419-021-03942-y)

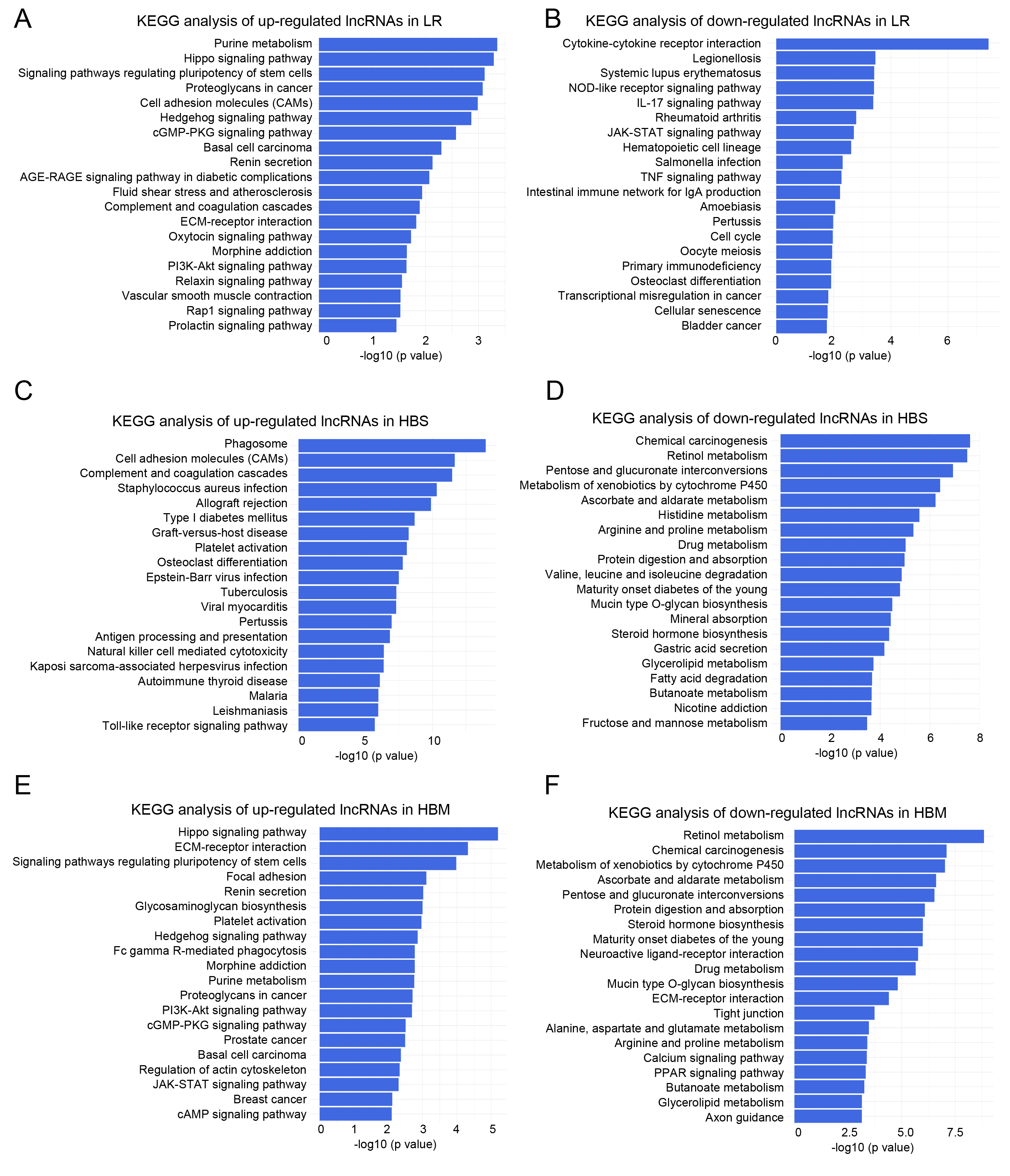

Supplement: Supplementary file 2 — Supplementary Figure 1 [file 41419_2021_3942_MOESM2_ESM.tif]

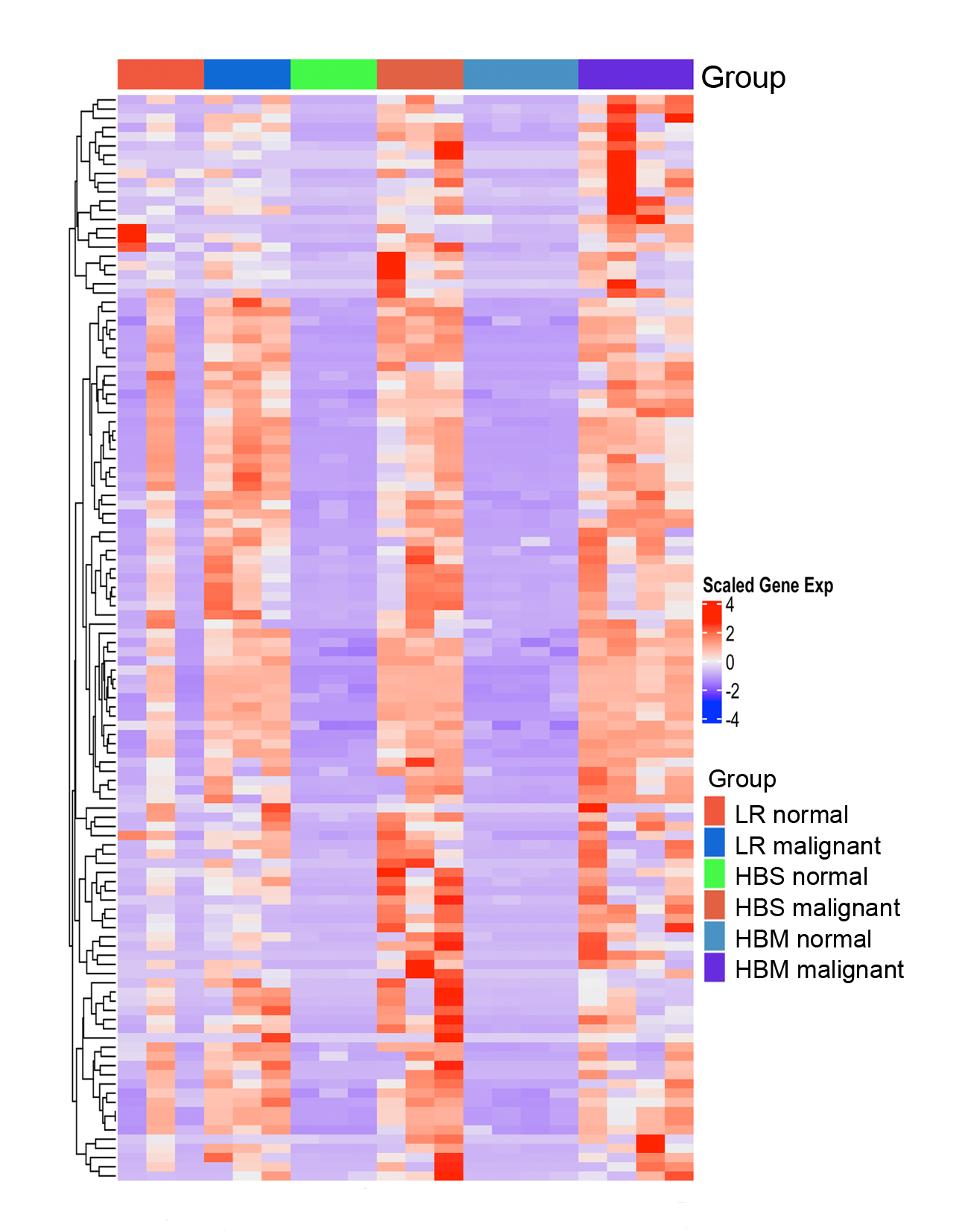

Supplement: Supplementary file 3 — Supplementary Figure 2 [file 41419_2021_3942_MOESM3_ESM.tif]

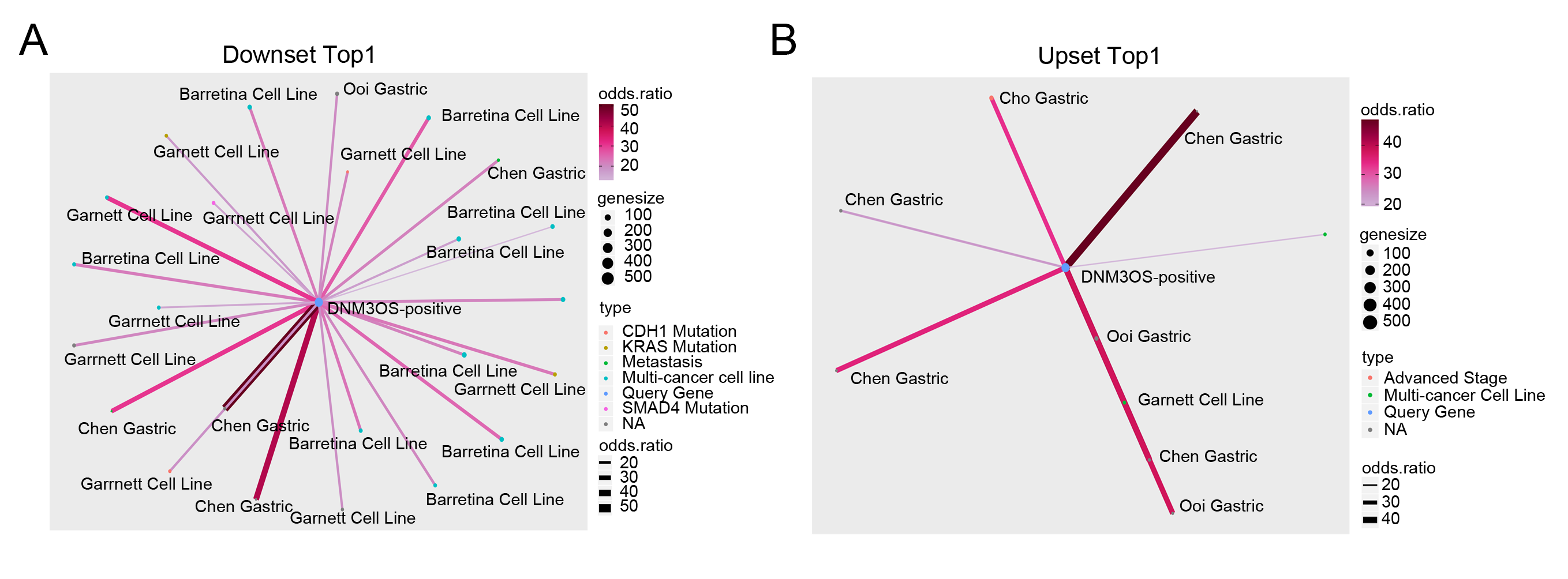

Supplement: Supplementary file 4 — Supplementary Figure 3 [file 41419_2021_3942_MOESM4_ESM.tif]

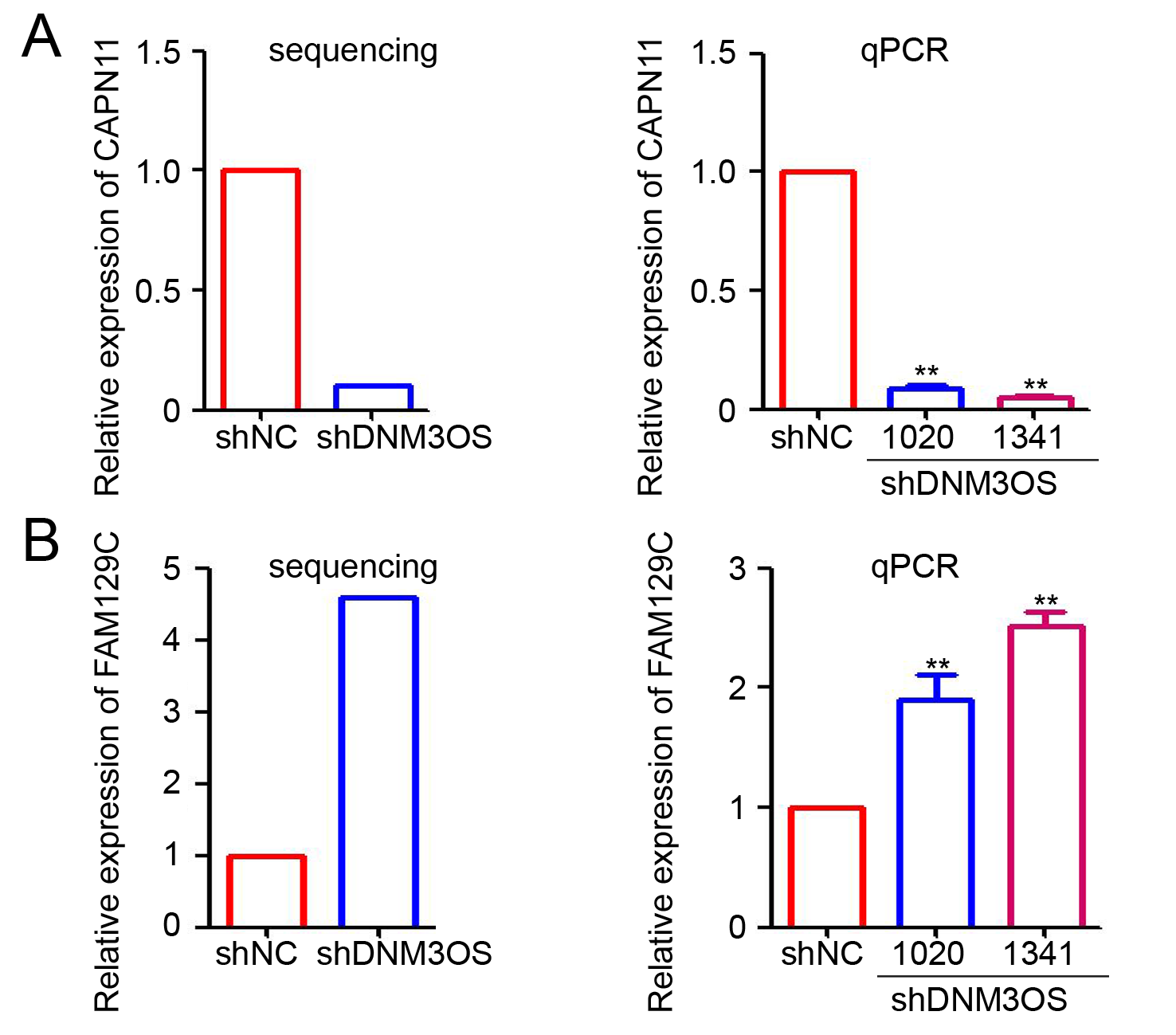

Supplement: Supplementary file 5 — Supplementary Figure 4 [file 41419_2021_3942_MOESM5_ESM.tif]
