# Supplementary material for: Integrated analysis of long non-coding RNAs and mRNAs associated with malignant transformation of gastrointestinal stromal tumors
Source: Cell Death Dis. 2021 Jul 3;12(7):669. doi: 10.1038/s41419-021-03942-y (PMC8254811; doi:10.1038/s41419-021-03942-y)
Supplement: Supplementary file 6 — Supplementary Table 1 [file 41419_2021_3942_MOESM6_ESM.docx]

| Supplementary Table 1. Clinicopathologic features of the sequencing samples | | | | | | | |
| --- | --- | --- | --- | --- | --- | --- | --- |
| Patient No | Sample name | Age | Gender | Tumor size in largest dimension (cm) | Mitotic count  (per 5cm^2^) | Risk stratification * | Mutation |
| 1 | LS1 | 60 | Male | 4.1 | 1 | Low | NA |
| 2 | LS2 | 50 | Female | 4.0 | <5 | Low | NA |
| 3 | LS3 | 47 | Male | 2.5 | 4 | Low | KIT exon 11 (P577_Y578insFP) |
| 4 | HBM1 | 63 | Female | 4.0 | 11 | High | KIT exon 11 (D580_K581insNPTQLPYDH) |
| 5 | HBM2 | 51 | Male | 4.2 | 24 | High | NA |
| 6 | HBM3 | 51 | Male | 4.9 | 13 | High | NA |
| 7 | HBM4 | 52 | Female | 2.0 | 14 | High | KIT exon 11 (W557_K558del) |
| 8 | HBS1 | 46 | Female | 10.5 | 4 | High | PDFGRA exon18 (I843_D846del) |
| 9 | HBS2 | 50 | Female | 11.0 | <5 | High | KIT exon 11 (V560D) |
| 10 | HBS3 | 66 | Female | 11.3 | 3 | High | NA |
| Note: LS: low risk stratification; HBM: high risk stratification based on mitotic count; HBS: high risk stratification based on tumor size; NA: not available.  *Risk classification was based on the modified NIH risk stratification. | | | | | | | |
