# Supplementary material for: Integrated analysis of long non-coding RNAs and mRNAs associated with malignant transformation of gastrointestinal stromal tumors
Source: Cell Death Dis. 2021 Jul 3;12(7):669. doi: 10.1038/s41419-021-03942-y (PMC8254811; doi:10.1038/s41419-021-03942-y)
Supplement: Supplementary file 7 — Supplementary Table 2 [file 41419_2021_3942_MOESM7_ESM.docx]

| S Table 2. Upregulated LncRNAs involved in Hippo signaling pathway. | | | | | | | |
| --- | --- | --- | --- | --- | --- | --- | --- |
| LncRNA | HBS | | |  | HBM | | |
|  | basemean | foldchange | p value |  | basemean | foldchange | p value |
| H19 | 47385.44 | 7.71978 | 0.002098 | | 48891.86 | 7.63734 | 0.00153 |
| DNM3OS | 1335.56 | 5.30474 | 0.019911 | | 1705.35 | 5.46514 | 0.01388 |
| DPP10-AS1 | 546.21 | 5.25036 | 0.020336 | | 288.36 | 4.77744 | 0.02443 |
| PRKCQ-AS1 | 306.77 | 4.43361 | 0.040685 | | 339.79 | 4.75845 | 0.02476 |
| ZFHX4-AS1 | 203.78 | 6.71875 | 0.006152 | | 295.65 | 6.73566 | 0.00401 |
| AC010980.2 | 144.61 | 5.41594 | 0.020193 | | 216.69 | 6.09564 | 0.00718 |
| SOCS2-AS1 | 79.28 | 5.00695 | 0.030911 | | 90.77 | 5.74816 | 0.01425 |
| FENDRR | 75.71 | 5.62845 | 0.017254 | | 33.34 | 5.54217 | 0.01986 |
| IGF2-AS | 62.21 | Inf | 0.001256 | | 136.42 | Inf | 0.00017 |
| MRPL23-AS1 | 61.73 | Inf | 0.001294 | | 31.79 | Inf | 0.00167 |
| LINC01096 | 42.34 | 7.93579 | 0.004976 | | 26.93 | Inf | 0.00203 |
| MEIS1-AS3 | 19.19 | 4.88002 | 0.047479 | | 109.66 | 6.19490 | 0.01592 |
