# Supplementary material for: Integrated analysis of long non-coding RNAs and mRNAs associated with malignant transformation of gastrointestinal stromal tumors
Source: Cell Death Dis. 2021 Jul 3;12(7):669. doi: 10.1038/s41419-021-03942-y (PMC8254811; doi:10.1038/s41419-021-03942-y)
Supplement: Supplementary file 8 — Supplementary Table 3 [file 41419_2021_3942_MOESM8_ESM.docx]

| Table 3. Association between DNM3OS expression and the clinicopathological characteristics of gastric GIST patients | | | |
| --- | --- | --- | --- |
| Variables | DNM3OS expression | | P value |
|  | Low expression (n=143) | High expression (n=108) |  |
| Gender (n, %) | | | |
| Male | 51 (35.7) | 54 (50.0) | - |
| Female | 92 (64.3) | 54 (50.0) |  |
| Age (years), mean ± SD | 58.73±11.05 | 57.58±11.55 | 0.427 |
| Tumor size (cm), mean ± SD | 5.96±3.16 | 7.53±5.15 | 0.003 |
| Mitotic count (n, %) | | | |
| ≤5/50 HPFs | 103 (72.0) | 48 (44.4) | <0.001 |
| 6-10/50HPFs | 24 (16.8) | 29 (26.9) |  |
| >10/50HPFs | 16 (11.2) | 31 (28.7) |  |
| NIH risk classification (n, %) | | | |
| Very low to low | 63 (44.0) | 24 (22.2) | <0.001 |
| Intermediate | 50 (35.0) | 31 (28.7) |  |
| High | 30 (21.0) | 53 (49.1) |  |
| Mutational status (n=130, %) | | | |
| KIT exon 9/11 | 60 (77.9) | 51 (96.2) | 0.015^a^ |
| PDGFRA exon 18 | 9 (11.7) | 1 (1.9) |  |
| WT | 8 (10.4) | 1 (1.9) |  |
| Prior TKI treatment (n, %) | | | |
| Yes | 53 (37.1) | 48 (44.4) | 0.245 |
| No | 90 (62.9) | 60 (55.6) |  |
| ^a^ Fisher exact test was used. | | | |
